# The translation of lipid profiles to nutritional biomarkers in the study of infant metabolism

**DOI:** 10.1007/s11306-017-1166-2

**Published:** 2017-01-28

**Authors:** Animesh Acharjee, Philippa Prentice, Carlo Acerini, James Smith, Ieuan A. Hughes, Ken Ong, Julian L. Griffin, David Dunger, Albert Koulman

**Affiliations:** 1 MRC Elsie Widdowson Laboratory, Cambridge, UK; 2 Department of Biochemistry, University of Cambridge, Cambridge, UK; 3 Department of Paediatrics, University of Cambridge, Cambridge, UK; 4 School of Food Science and Nutrition, University of Leeds, Leeds, UK; 5 MRC Epidemiology Unit, University of Cambridge, Cambridge, UK; 6 NIHR BRC Clinical Metabolomics and Lipidomics Laboratory, Level 4, Laboratory Block, Cambridge University Hospitals, University of Cambridge, Hills Road, Cambridge, CB2 0QQ UK

**Keywords:** Lipidomics, Biomarker discovery, Random Forest, Infant nutrition

## Abstract

**Introduction:**

Links between early life exposures and later health outcomes may, in part, be due to *nutritional programming* in infancy. This hypothesis is supported by observed long-term benefits associated with breastfeeding, such as better cognitive development in childhood, and lower risks of obesity and high blood pressure in later life. However, the possible underlying mechanisms are expected to be complex and may be difficult to disentangle due to the lack of understanding of the metabolic processes that differentiate breastfed infants compared to those receiving just formula feed.

**Objective:**

Our aim was to investigate the relationships between infant feeding and the lipid profiles and to validate specific lipids in separate datasets so that a small set of lipids can be used as nutritional biomarkers.

**Method:**

We utilized a direct infusion high-resolution mass spectrometry method to analyse the lipid profiles of 3.2 mm dried blood spot samples collected at age 3 months from the Cambridge Baby Growth Study (CBGS-1), which formed the discovery cohort. For validation two sample sets were profiled: Cambridge Baby Growth Study (CBGS-2) and Pregnancy Outcome Prediction Study (POPS). Lipidomic profiles were compared between infant groups who were either exclusively breastfed, exclusively formula-fed or mixed-fed at various levels. Data analysis included supervised Random Forest method with combined classification and regression mode. Selection of lipids was based on an iterative backward elimination procedure without compromising the class error in the classification mode.

**Conclusion:**

From this study, we were able to identify and validate three lipids: PC(35:2), SM(36:2) and SM(39:1) that can be used collectively as biomarkers for infant nutrition during early development. These biomarkers can be used to determine whether young infants (3–6 months) are breast-fed or receive formula milk.

**Electronic supplementary material:**

The online version of this article (doi:10.1007/s11306-017-1166-2) contains supplementary material, which is available to authorized users.

## Introduction

Early nutritional biomarkers are essential in the study of infancy metabolism, and for investigations into ‘metabolic programming’. This concept is based on increasing evidence that nutrition and growth in early life are linked to long-term health outcomes across the life course (Horta and Victora 2013; Singhal 2006). Importantly, the underlying physiological and metabolic mechanisms are poorly understood and difficult to study. This is due to a lack of relevant tools that encompass both the precision and resolution required to investigate the differences in the endogenous metabolic responses of infants to breast or formula milk. In addition, there is the necessity for such tools to be minimally invasive thus limiting ethical concerns in terms of obtaining suitable samples from infants. Furthermore, it is also essential that newly found differentiating metabolite signals from lipids are validated so that they can be recognised as reliable biomarkers (Strimbu and Tavel 2010; Issaq et al. 2011; Willis and Lord 2015; Xia et al. 2013).

Biomarker discovery has emerged as a rapidly growing field and in the last 3 years and there have been more peer-reviewed reports published (ca. 16,000) than in all the years before combined (ca. 13,000) (http://www.scopus.com). All this work has led to discovery of many differentiating lipids from an increasing number of papers on metabolomics (Koulman et al. 2014) and shotgun lipidomics (Ivanova et al. 2009; Shevchenko and Simons 2010; Wolf and Quinn 2008), many of which show very relevant and significant differences in lipid profiles between different pathologies, populations and diets. However, many of these results are rarely validated in independent studies, and this is essential in enabling the translation from finding differential lipid profiles to the application of lipid biomarkers.

We developed and validated a lipid profiling method using high resolution mass spectrometry to determine the lipid composition of dried blood spot samples (Koulman et al. 2014). We applied this method to samples from the Cambridge Baby Growth Study (CBGS) to determine if infant nutrition had a significant impact on lipid metabolism and infant growth. We reported the significant differences between the lipid profiles of breast-fed and formula-fed infants (Prentice et al. 2015). Our aim was to validate these differences in the lipid profile between breastfed and formula-fed infants and be able to call these lipids biomarkers of infant nutrition.

## Methods

### Cohort study design

The Cambridge Baby Growth Study (CBGS) is a prospective observational birth cohort, focussing on the antenatal and early postnatal determinants of infancy growth (Prentice et al. 2015). Mothers were recruited during early pregnancy from a single antenatal centre in Cambridge, between 2001 and 2009. Their infants were seen at birth by trained research nurses and followed-up at 3 and 12 months respectively with detailed anthropometry. Dried blood spots (DBS) were collected using capillary heel prick sampling, dropping blood spots onto WhatmanTM^®^ untreated filter paper (Ahlstrom 226, ID Biological Systems). Infancy feeding (exclusive breast-, mixed or exclusive formula-feeding) was assessed by questionnaire at age 3 months. The study was approved by the Cambridge research ethics committee and all mothers gave written consent. With slight modifications to the protocol, two further waves of data collection were subsequently completed (labelled CBGS2 and POPS).

#### Cambridge Baby Growth Study dataset 1 (CBGS-1)

We modified our direct infusion high-resolution mass spectrometry (HRMS) method with plasma/serum samples and applied this initially to 241 DBS samples (dataset 1) from the Cambridge Baby Growth Study (CBGS) cohort, a prospective observational birth cohort. The samples were taken at 3 months (m) of ages when infants also had detailed anthropometrical measurements, and feeding practice was assessed by questionnaire (exclusive breastfeeding (HM), exclusive formula-feeding (FM), or mixed feeding (HM&FM). Dataset-1 consisted of a total of 239 infants at 3 months of age, who had different feeding practice and coded as following: 85 HM, 87 FM, 67 mixed-fed. Because of the substantial differences in the lipid profile between these groups (Prentice et al. 2015), we aimed to validate these findings in a new independent sample set, using these original data as a training dataset. The study was approved by the Cambridge local research ethics committee, and all mothers gave informed written consent (LREC 00/325).

#### Cambridge Baby Growth Study dataset 2 (CBGS-2)

A second lipidomics dataset (dataset-2) from the Cambridge Baby Growth Study cohort (CBGS-2) was available, which was based on samples that were obtained from infants born small for gestational age or from diabetic mothers. The total number of samples (n) were 95 (43 breast-fed, 25 formula-fed, 27 mixed-fed at 3 months). This dataset-2 was used for validation of the potential biomarkers found in dataset-1. The study was approved by the Cambridge local research ethics committee, and all mothers gave informed written consent (LREC 11/EE/0068).

#### Pregnancy Outcome Prediction Study (POPS)

A third dataset was available consisting of infants at both 3 and 6 months of age (*n* = 40, 16 breast-fed, 11 formula-fed, 13 mixed-fed at 3 months). This dataset was from the end of CBGS recruitment when more detailed data collection had been added to the CBGS protocol, but the demographics of the cohort were very similar. In addition to recording feeding practice, data for these infants were available on volume of formula intake (ml per day) in both mixed- and exclusively formula-fed infants. This dataset-3 was used for validation of the potential biomarkers found in dataset-1. Ethical approval for the study was obtained from the Cambridgeshire 2 Research Ethics Committee (reference 07/H0308/163).

### Data generation

DBS on filter paper cards were air dried at ambient temperature overnight, before being stored in Ziploc storage bags at −20 °C until analysis, when a single 3.2 mm spot was punched from the larger DBS.

### High-resolution mass spectrometry (HRMS)

The large-scale lipidomics method was adapted for a platform developed for plasma samples (Eiden et al. 2015) to enable the analysis of DBS samples and has been previously described (Koulman et al. 2014). In brief, the blood/analytes from a 3.2 mm DBS was extracted with 100 µl of MilliQ H_2_O in a well of a glass-coated 2.4 ml deep well plate (Plate+TM, Esslab, Hadleigh, UK), then 250 µl of MeOH was added. Lipids were partitioned into 500 µl of Methyl-tertiary-butyl ether. After centrifugation, the organic layer was concentrated and used for lipid analysis. Samples were infused into a Thermo Exactive benchtop orbitrap (Hemel Hampstead UK), using an Advion Triversa Nanomate (Ithaca US) and data acquired in both positive (+1.2 kV) and negative (−1.5 kV) mode voltages. All experiments were run with blank controls and two different quality control samples. In total, 218 lipid signals could be detected robustly using this method. The lipids have been identified as described previously (Koulman et al. 2014) and the identification is at level 2 of the Metabolomics Standards Initiative .

All datasets are provided in the supplementary material 1.

### Data analysis strategies

**Fig. 1:**
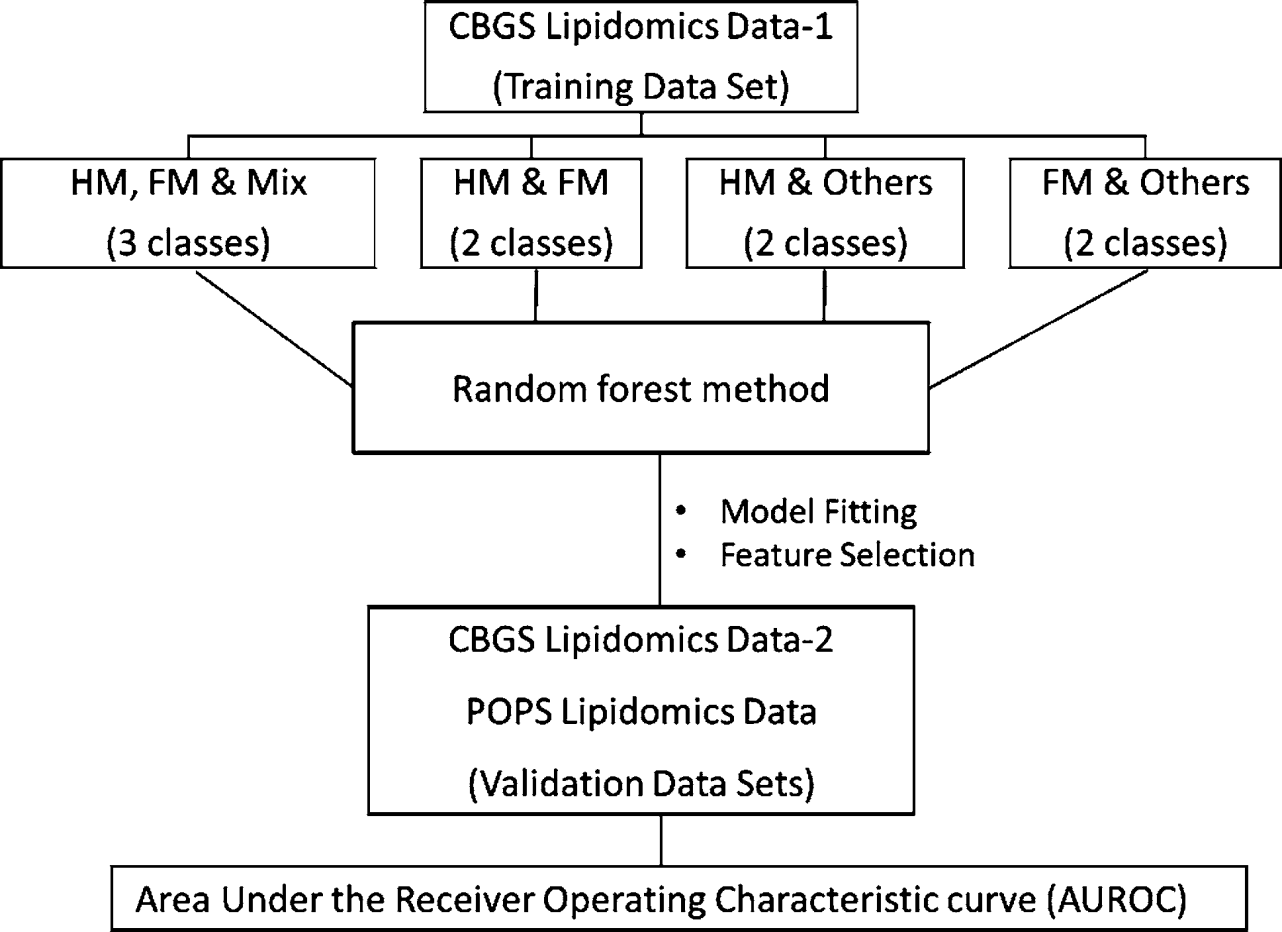
Workflow for the data analysis. Random Forest (RF) classification was used to select subsets of lipids from lipidomics data and different classes of milk nutrition are shown. CBGS-1 data were used as a training set whereas CBGS-2 and POPS data were used for validation and quantified using the area under a receiver operator characteristics (AUROC)

The lipid signals obtained were semi-quantitative, with the signal intensity of each lipid expressed relative to the total lipid signal intensity, for each individual, per thousand. Raw HRMS data were processed using XCMS (http://www.bioconductor.org) and Peak picker v2.0 (an in-house R script). All lipid species where >30% of cases had a value of zero, were excluded from further analyses. A detailed data analysis diagram is shown in Fig. 1.

Lipidomics data were generated from DBS of three studies: CBGS-1, CBGS-2, and POPS. For the CBGS-2 and POPS studies, there were DBS samples collected at 3 months and 6 months. For all participants, the diet was recorded and coded as follows: human milk (HM), formula milk (FM) and mix (formula and human milk, HM & FM). Diets were considered as different class or groups and hence the classification method was appropriate. Within CBGS-2 and POPS datasets, the total daily amount of milk (ml) were also assessed.

### Random Forest

Random Forest (RF) (Breiman 2001; Acharjee et al. 2011, 2016)), a machine learning ensemble method was employed in conjunction with multiple learning algorithms to obtain better predictive performance. This included a backwards elimination (Diaz-Uriarte and Alvarez de Andres 2006).

This allowed all the metabolite data to be combined in a nonlinear way rather than solely in a linear way and hence allow the discovery of more complex dependencies. RF was used for both classification and regression-based analysis (Breiman 2001) and involved a bootstrapping method for training or testing and decision trees for prediction. Bootstrapping generates random samples from the dataset with replacement. Every bootstrapped sample has a corresponding left out or ‘out-of-bag’ (OOB) sample that is used to test the algorithm. For example, if we generate 100 bootstrapped samples, every time we get a set of predictions from the training samples. The final prediction is simply the average of all 100 predictions from the trees that do not contain training samples in their respective bootstrap sample (test samples).

#### Random Forest classification

Random Forest was exploited as a multiclass classification method to classify different types of feeding (HM, FM or combinations). For the classification model, RF needed to use some of the parameters to be set “*a priori”*. For example, the number of trees (ntree) and the number of variables (lipids) randomly sampled as candidates at each split (mtry) needed to be defined. We used ntree = 500 and mtry = square root of the number of variables in our models. For example, for this dataset, mtry was set to 15 and the nearest integer to the square root of 218, the total number of metabolites.

#### Random Forest regression

Our aim was to assess if the significant differences in the lipid profile of infants due to their early nutrition could indeed be replicated. Again RF was employed to identify the lipids with the strongest predictive power in the discovery cohort and subsequently we tested the chosen lipids in two different datasets for validation.

In regression analyses, RF was used to predict the total amount of quantified milk from CBGS-2 and POPS data sets and construct a predictive model for each of the response clinical phenotypes, whilst quantifying the importance of each variable. Quantification was based on the variation explained (Q^2^) by the model, not only as a measure of goodness of fit of the data, but also determined by left-out “out-of-bag” samples as a proxy for predictive quality. The variance explained in RF is defined as 1.0 − (mean square error (MSE)/Q^2^), where MSE is the sum of squared residuals of the OOB samples divided by the OOB sample size. RF regression also requires parameters for example number of trees (ntree), and the number of variables (lipids) randomly sampled as candidates at each split (mtry). In this work, ntree = 500 and mtry = 72 approximately one-third of 218.

#### The backward elimination method

To select metabolites Random Forests were fitted iteratively and at each iteration a new forest was built after discarding 20% of the metabolites with the smallest variable importance. The selected set of metabolites was then fitted with the RF model to check the OOB error rate. This procedure was repeated using the varSelRF function from varSelRF package in R-CRAN (Diaz-Uriarte and Alvarez de Andres 2006).

#### Permutation test

RF quantifies the importance of lipids that classify the different nutritional intakes but does not give a significance level or a threshold to choose a possible subset of associated metabolites. Therefore, a permutation test was included to indicate significance of the lipid’s associations in this study. In this situation, the different feeding categories were randomized, each time applied to the RF 1000 times for 1000 different randomizations. In each analysis, the Q^2^ and variable importance in terms of a decrease in node impurities were estimated. Node purity values were ordered from the permuted datasets and the 95th percentile of the distribution node impurity values were taken to assess the significance of the individual lipids. The same was done for the 95th percentile of Q^2^ to denote significance of the Q^2^ value in RF regression model.

### Software

All statistical analysis was done in using R software (v3.3.0). Two R packages for Random Forest analysis: Random Forest (Liaw and Wiener 2002) and varSelRF (Diaz-Uriarte and Alvarez de Andres 2006) were required. To aid reproducible research, the R- Scripts are provided in the supplementary material 2.

## Results

### Classification

#### Results from CBGS Dataset −1

RF classification method was applied in the following four different ways. In each situation, we estimated the percentage of out-of-bag (OOB) error using all and selected variables.

HM, FM, and *Mix* (three-way classification): In this situation, HM, FM and HM & FM were considered as three classes. The OOB class error from RF classification method using all variables was 28% and after selected variable was 27%.

HM and FM (two-way classification): In this situation, HM and FM were treated as two classes. The OOB error was estimated as 3.5% using all variables whereas after selection of variables, it was 2.8%.

HM and *Others* (two-way classification): In this situation, HM feed samples were treated as one class and rest of other samples were considered as one class. The OOB error was estimated 18% using all variables, whereas, after selection of variables, it was 17%.

FM and *Others* (two-way classification) analysis: In this situation, FM feed samples were treated as one class and rest of other samples were considered as one class. The OOB error was estimated 8.7% using all variables whereas after selection of variables, it was 10%.

**Fig. 2:**
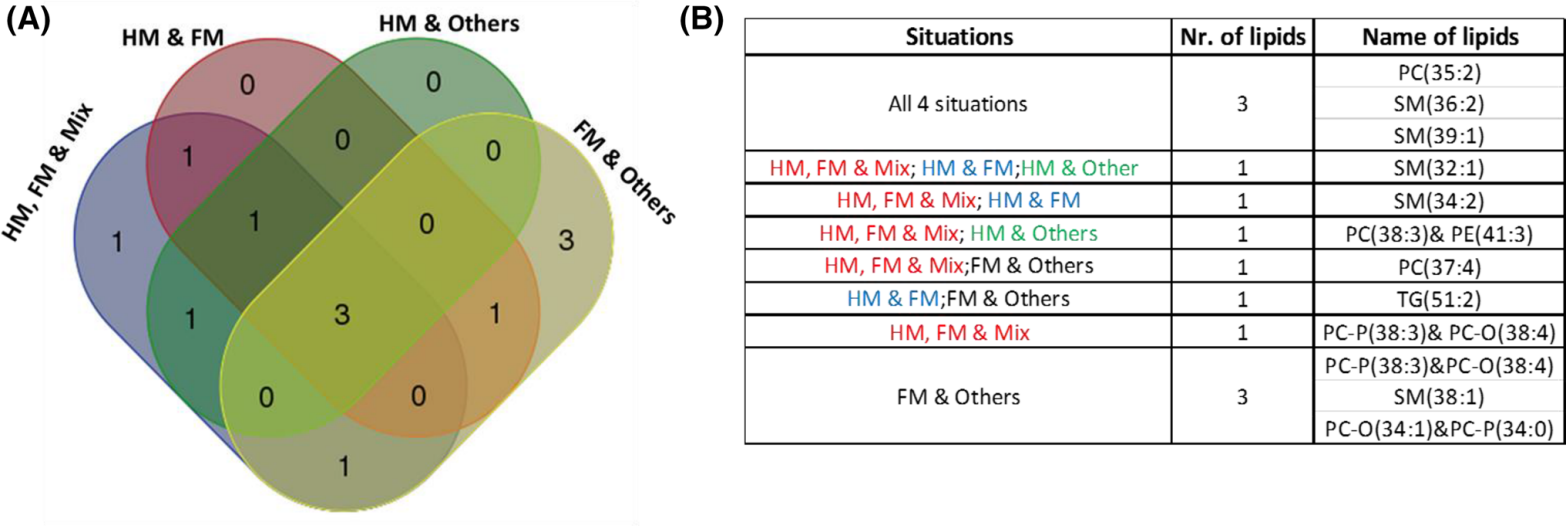
Lipids selected using backwards elimination process. **a** shows common and unique lipids in the different situations. **b** lists the lipids associated with the situations explored. For simplicity, the situations are marked in *different colours*

Using a backwards elimination method, we were able to identify the lipids that had the strongest impact on the classification. The list of selected lipids is shown with their uniqueness or overlap for their ability to predict class membership in the different situations (Fig. 2).

#### Validation

**Fig. 3:**
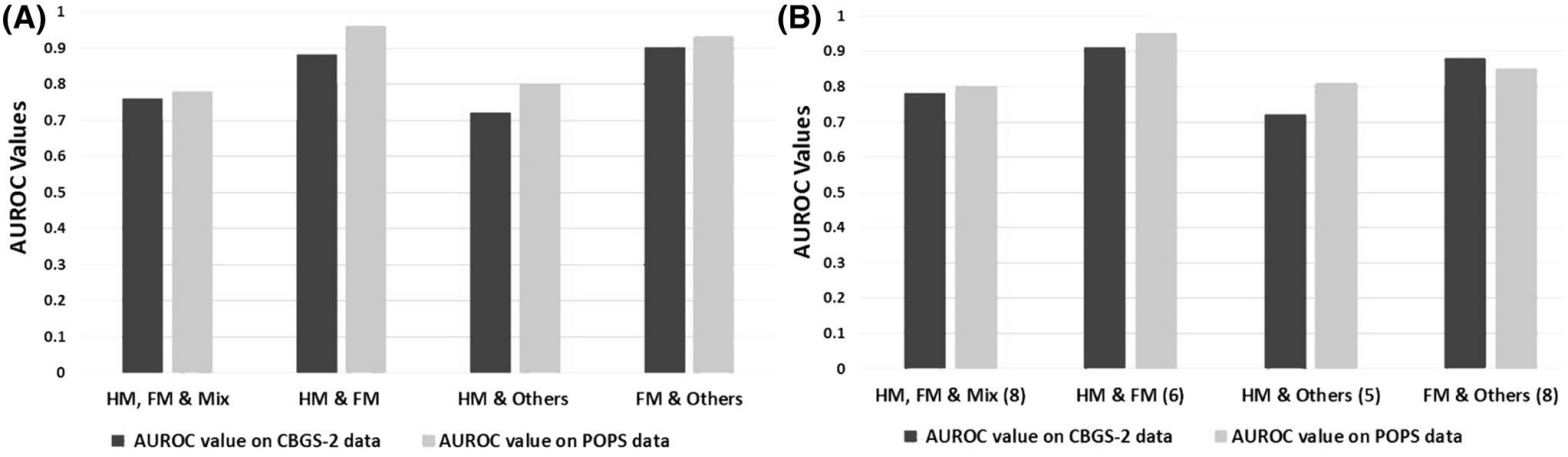
Summary of the area under receiver operating characteristic (AUROC) curves in different situations with human milk (HM), HM & formula (Mix) and formula (FM). Four situations are described with all lipids in (**a**) and selected lipids in (**b**) and their impact on AUROC values are summarised clearly showing that the selected lipids are enough to predict in both CBGS-2 and POPS datasets

RF classification was applied to the dataset-1 (treated as a training set) to identify the differentiating lipids, which we aimed to translate to biomarkers through the validation in datasets-2 and dataset-3. The selected lipids were applied to the CBGS-2 and POPS datasets, as validation work and to check the area under receiver operating characteristic (AUROC) performance. A summary of the results is shown in the Fig. 2(a) and (b). Figure 3 shows a summary of the AUROC curves for the test and validation sets. Figure 3(a) represents AUROC values in four different situations using all the lipids whereas 3(b) representing AUROC performance with selected lipids from training data (CBGS-1) set. Using backward elimination, we were able to filter lipids out of the entire lipid pool (p = 218) in all the other situations and from the Fig. 3(b). Three lipids PC(35:2), SM(36:2) and SM(39:1) were found to be common in all the situations (HM, FM vs. *Mix*; HM vs. FM; HM vs. *Others* and FM vs. *Others*) from dataset-1. It is clear that selected lipids do not compromise the AUROC values in comparison to the results obtained with all the lipids (p = 218) on CBGS-2 (dataset-2) and POPS data (dataset-3). This is because of elimination of lipids that were not relevant for the predicting the response variable. Validation models were also created using the 6-month infancy data available (POPS), and can be found in the Supplementary Material 3.

### Prediction of volume of formula milk using regression analysis

**Fig. 4:**
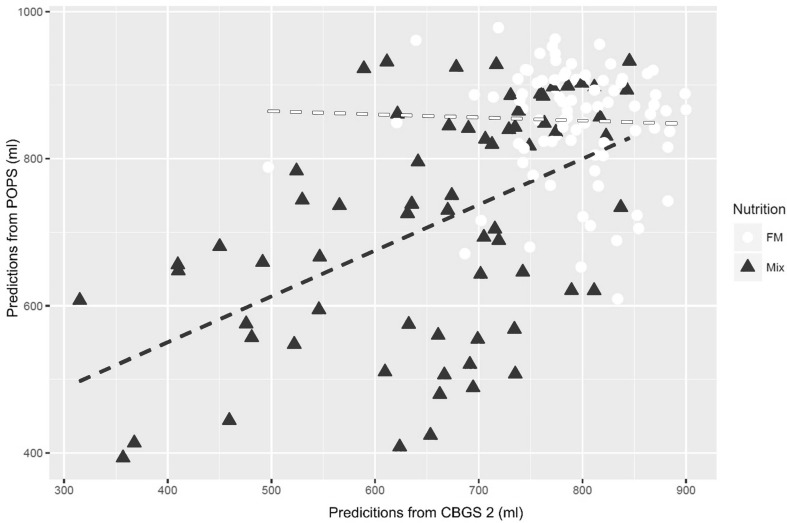
Predictions of the volume of formula milk (ml) in the CBGS-1 samples (FM and mix samples only) from CBGS-2 and POPS dataset separately using selected lipids. The *dashed lines* show the relationships within FM (*red*) and Mix feed (*blue*) samples. The FM showed a limited correlation with two predictions whereas Mix feed samples show a linear relationship with Pearson correlation 0.56

Using regression models, we aimed to predict the daily intake volume of formula milk (ml) in the dataset-1 from dataset-2 and dataset-3 respectively. The variation explained (Q^2^) by CBGS-2 and POPS were 32 and 51% respectively using all the lipids. The Pearson correlation (r) value of the correlation between these two predictions was 0.65 (p < 10^−5^) (Supplementary Material 4). Furthermore, we selected 13 significant lipids from POPS data based on the backwards elimination approach and used only them to predict the amount of the volume of formula milk (ml) in the dataset-1. The variation explained (Q^2^) in CBGS-2 and POPS were 38 and 65% respectively (Fig. 2) and Pearson correlation (r) value from two predictions using all the samples (in the HM, FM and *Mix* situation) was 0.87 (p < 10^−6^). The predictions of the amount of the volume of formula milk (ml) in the CBGS-1 from CBGS-2 and POPS dataset using selected lipids are shown in Supplementary Material 5. The 13 selected lipid groups were: LysoPC(14:0), DG-H_2_0(33:1), SM(36:2), PC(34:2), SM(39:1), PC(37:4), PE(40:3) with PC(37:3), TG(49:1), PE(43:4), TG(51:3), TG(51:2), TG(53:3) with TG(53:2) and finally sphingomyelins SM(36:2) and SM(39:1) already shown to be robust and predictive nutritional biomarkers. The daily milk intake volume in the CBGS-2 and POPS datasets provided the opportunity to predict the amount of milk in the CBGS-1 dataset. In Fig. 4, we show how formula milk (FM) and Mix samples are distributed, the FM samples form a defined cluster, whereas the Mix samples are dispersed around them.

## Discussion

The study of lipid metabolism in healthy infants remains largely unexplored. One of the main reasons is that repeat drawing of blood from healthy newborns is regarded as over-invasive. Dried blood spots (DBS) from heel pricks have long been established as the most appropriate sample format and this is now commonly applied to screen infants for inborn errors of metabolism (Chace et al. 1993; Pandor et al. 2004). Until recently, methods were not sensitive enough to discriminate small differences in circulatory-metabolic responses of healthy infants. We have developed and validated a lipid profiling method using DBS from infants (Koulman et al. 2014). The method provides data on approximately 200 endogenous lipids from a single 3.2 mm DBS disc. The process is automated and fast, very robust, inexpensive and therefore suitable for studying large cohorts. We applied this method to a subset of DBS samples collected in the Cambridge Baby Growth Study and showed that lipid profiles of breastfed infants are significantly different to those of formula-fed infants (Prentice et al. 2015). This work led to the discovery of specific blood lipid signals that can indicate whether a 3-month-old infant has received either breastfeeding or formula feeding.

We showed in the original discovery study only the discrimination between exclusively breastfed and exclusively formula fed infants (Prentice et al. 2015). However, there is a considerable number of infants that receive a mixture of breast milk and formula (mixed-feeding). We expected that it would be possible to predict the amount of formula milk mixed-fed infants received, based on the infants’ lipid profile, enhancing the application of lipid biomarkers in early life. By expanding this work across two more datasets we were able to validate the putative lipid biomarkers of infant nutrition. We used a reproducible strategy to investigate lipidomics data and reveal lipids for different diets and also validate these as biomarkers in two independent data sets. We used Random Forest throughout our analysis both in classification and regression mode. Furthermore, we selected lipids iteratively that resulted in a small class errors in the classification and a higher Q^2^ (variation explained) in the regression.

Dataset-2 and dataset-3 were available for independent validation and provided an opportunity compare the lipids identified from the dataset 1. We found PC(35:2), SM(36:2) and SM(39:1) in dataset-1 with a discriminatory power and these were validated in dataset-2 and dataset-3. The response vector (for both class and quantification of formula feed) was randomized 1000 times to investigate the robustness of the model.

The three key lipids that distinguished breastfed from formula fed infants are the phosphatidylcholine PC(35:2) and two sphingomyelins SM(36:2) and SM(39:1). Furthermore, two of the three biomarkers contain odd chain fatty acids and these two biomarkers are not correlated, which suggest that the origins of the odd chain fatty acids for these two lipids are different. The vast majority of lipids in both breast milk and formula milk are triglycerides (ca 98%), while triglycerides are a much smaller part of the blood lipids in the infant. Therefore, it cannot be assumed that all the lipids found in the blood are directly coming from the diet. An exception could be SM(39:1), which is higher in the formula-fed infants. SM(39:1) is mainly SM(16:1/23:0) based on fragmentation data. It has been shown that the SM(39:1) is one of the common long chain sphingomyelins in different formula milks (Fong et al. 2013) and not commonly present in breast milk (Blaas et al. 2011). Total sphingomyelin content in breast milk is about a five-fold higher than in formula milk, which could partly explain why SM(36:2) is higher in breastfed infants. However, not all sphingomyelins are significantly higher in breastfed infants and it is likely that lipid homeostasis curbs differences in sphingomyelin intake. For PC(35:2) (mainly PC(17:0/18:2)) it is unclear why this phospholipid is increased due to breastfeeding. Both breast milk and formula contain odd chain fatty acids but there is no literature that has quantified the odd chain fatty acids comprehensively. It is possible that breast milk consumption leads to an increased endogenous production of odd chain fatty acids due to alpha-oxidation (Jenkins et al. 2015).

The lipids that can predict the amount of formula intake are to a large extend odd chain fatty acid containing lipids, such as SM(39:1), TG(51:2), TG(53:2) etc. Although breast milk contains odd chain fatty acid, the level of very long odd chain fatty acids, such as C23:0 in SM(39:1) suggests that is a direct marker of formula intake. This work clearly indicates that the lipid metabolism is significantly impacted by nutrition very early during child development and the lipids identified can be used to predict whether or not the infant has received formula feed and, if so, how much.

In terms of comparing Random Forest to other similar approaches, (Scott et al. 2013) studied and tested 28 classifiers on data from NMR and mass spectroscopy from different origins as the training set for a number of multivariate tools. Data came from four metabolomics or food projects, where the numbers of classes differed. Random forests were found to perform consistently the best on high-dimensional data but only reported in less than 5% of the research literature. As a high dimensional procedure importantly, where the number of metabolites (p) is much larger than the number of samples (n) it also has an internal cross-validation procedure using out of the box (OOB) sampling. A limitation, however, is that Random Forest will default to using all variables simultaneously and a pre-selection of the variables is recommended. Backward elimination is implemented in the package varSelRF (Diaz-Uriarte and Alvarez de Andres 2006) uses the OOB as minimization criterion, however, the improved performance achieved with the reduced model is likely to be biased. One possible solution is to apply a cross-validation-based protocol and (Tsamardinos et al. 2014). In this work, some lipids found were common to both the approaches whereas others were specific to the mode applied.

The result of this study shows that the lipid status markers are reproducible. Their reliability in predicting the exposure of formula intake *vs*. breast milk requires further investigation and will be of great value to future epidemiological and public health research into the effect of mixed-feeding strategies.

### Concluding remarks

We present a reliable strategy for prioritising putative biomarkers from lipidomics and show the lipid profile differs due to nutrition in infants. Differences were further studied in two validation datasets which allowed the validation of unambiguous lipid biomarkers. This work was able to identify and confirm that three lipids: PC(35:2), SM(36:2) and SM(39:1) can together be used as robust biomarkers of infant nutrition.

## Electronic supplementary material

Below is the link to the electronic supplementary material.

Supplementary material 1 (XLSX 980 KB)

Supplementary material 2 (TXT 4 KB)
